# Correction to: Performance of Blood-Based Nucleocapsid Antigen Tests for Diagnosis of Severe Acute Respiratory Syndrome Coronavirus 2 Infection and Infectious Viral Shedding: A Systematic Review

**DOI:** 10.1093/ofid/ofad445

**Published:** 2023-08-22

After the publication of this article [Mathur S, So M, Tahir P et al. Performance of Blood-Based Nucleocapsid Antigen Tests for Diagnosis of Severe Acute Respiratory Syndrome Coronavirus 2 Infection and Infectious Viral Shedding: A Systematic Review. Open Forum Infectious Diseases, Volume 10, Issue 8, August 2023, ofad346, https://doi.org/10.1093/ofid/ofad346], the sentence ‘In nonsevere cases, there was an exponential decay in N-antigen concentration over time [22].’ was erroneously omitted from the first paragraph of the Discussion section and has been added back in. The updated version of this article is currently available online.

